# *QuickStats:* Percentage Distribution of Heat-Related Deaths, by Age Group — United States, 2024

**DOI:** 10.15585/mmwr.mm7534a3

**Published:** 2026-09-03

**Figure:**
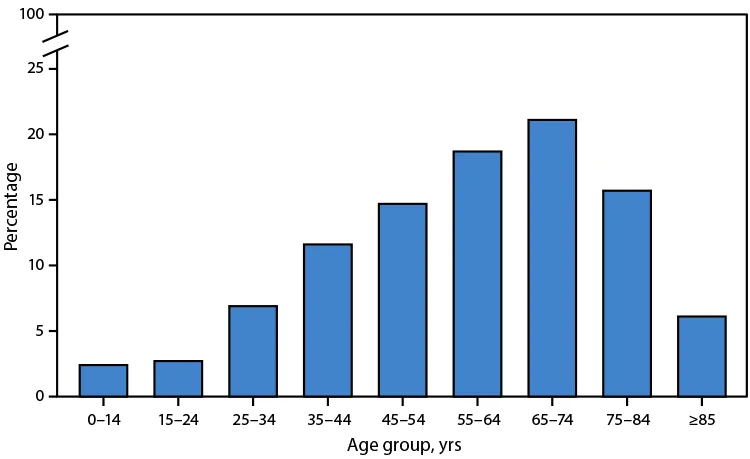
In 2024, a total of 2,391 heat-related deaths were reported in the United States. The percentage of heat-related deaths increased with age through ages 65–74 years, with the highest percentage (21.1%) reported among adults in this age group. The percentage of heat-related deaths then decreased through age ≥85 years.

**Supplementary Table:** https://stacks.cdc.gov/view/cdc/258541

**Source:** National Center for Health Statistics, National Vital Statistics System, Mortality Data, 2024

For more information on this topic, CDC recommends the following link: Heat & Health Tracker | CDC

